# Examining social support and procrastination among college students

**DOI:** 10.3389/fpsyg.2024.1425524

**Published:** 2024-08-29

**Authors:** Ashley Miller, Diamond Bravo, Elisha Arnold, Brenda Rincon, Carolyn Murray

**Affiliations:** ^1^Department of Psychology, University of California, Riverside, Riverside, CA, United States; ^2^Department of Applied Psychology, New York University, New York City, NY, United States

**Keywords:** procrastination, intolerance of uncertainty, social support, family support, friend support

## Abstract

The current study investigated how social support may mitigate the risk of procrastination, particularly among those high in *intolerance of uncertainty*. This study examined associations between personality traits, procrastination, and perceived social support among 394 undergraduate students. Participants completed self-reported measures of intolerance of uncertainty, procrastination, and social support from family, friends, and significant others. Regression analyses revealed a significant interaction between family social support and intolerance of uncertainty in predicting procrastination. Study findings have implications for understanding how familial support resources may reduce risks for procrastination in college students.

## Introduction

*Procrastination* is defined as a “voluntarily delay[ing] an intended course of action, despite expecting to be worse off for the delay” (Steel, 2007, p. 66). Throughout each academic term, college students are faced with multiple tasks, such as preparing for exams and completing term papers. These tasks require substantial preparation (e.g., studying, drafting papers) that individuals commonly delay. Procrastination, or the tendency to delay the completion of tasks, is generally considered a negative behavior, because delaying on tasks often results in poorer job performance (Metin et al., 2018). Theoretical works posit that procrastination, at its core, is an issue concerning both self-regulation (Steel, 2007) and emotion regulation (Pychyl and Sirois, 2016). For example, students may procrastinate because they struggle to regulate their behavior, such as choosing a time to study and following through with that goal. Alternatively, students can struggle with anxiety or stress they feel toward a particular task, and then cope with anxiety by avoiding the task (e.g., procrastinating) to improve their mood temporarily. Students who are high in personality traits that predispose them to negative affect, such as intolerance of uncertainty, are more at risk of procrastination (Fourtounas and Thomas, 2016). Despite growing knowledge about risk factors that increase the likelihood of procrastination, limited research and theorizing has considered how protective factors, such as social support, may buffer against procrastination among college students. This study examines how various sources of social support (i.e., family, friend, and/or people who are special to the individual) may buffer the relationship between intolerance of uncertainty (i.e., anxiety and/or stress) and procrastination.

Procrastination is especially prevalent in college students. Approximately 75% of students procrastinate, compared to 15–20% of the general population (Steel, 2007). Some students have reported using procrastination as a strategy to increase their motivation; by putting off tasks until the last minute, they induce deadline-related stress that motivates them to complete the task (Schraw et al., 2007). However, the effectiveness of this strategy is unknown. The harmful consequences of procrastination in student populations are well documented; students who procrastinate tend to report lower GPAs (Gareau et al., 2019). Indeed, prior reviews indicate that procrastination is consistently related to lower final exam scores and assignment grades (Steel, 2007). To avoid procrastination in college students, it is worth examining how specific protective factors, such as various forms of social support, may buffer the relationship between procrastination and risk factors for procrastination, such as intolerance of uncertainty.

Procrastination is a many-sided problem (Mann, 2016), involving multiple cognitive, affective, and behavioral factors and subsequently, there are a variety of reasons why college students avoid working on academic tasks in a timely manner. This discussion focuses on one aspect in particular, *intolerance of uncertainty*. Intolerance of uncertainty refers to an individual’s tendency to view the mere possibility of a negative event as unacceptable and threatening, regardless of the likelihood of its occurrence (Carleton et al., 2007). This psychological construct is characterized by a heightened sensitivity to uncertain or ambiguous situations, leading to increased levels of anxiety, stress, worry, and overall negative affect (Anderson et al., 2019). Experiencing negative affect, induced by intolerance of uncertainty, such as stress, is related to increased procrastination, particularly among individuals who attempt to use procrastination as a mood-regulations strategy (Sirois, 2023), making intolerance of uncertainty a risk factor that is associated with greater likelihood, or increased incidence of, procrastination.

### Unpacking intolerance of uncertainty

High intolerance of uncertainty is associated with more procrastination (Fourtounas and Thomas, 2016). Broadly, uncertainty is an uncomfortable experience that is tied to one’s emotional state (Gu et al., 2020). Individuals who report difficulty tolerating uncertainty, a personality trait known as intolerance of uncertainty, tend to view the mere possibility of a negative event as unacceptable and threatening, regardless of the likelihood of its occurrence (Carleton et al., 2007). This psychological construct is characterized by a heightened sensitivity to uncertain or ambiguous situations, leading to increased levels of anxiety, stress, worry, and overall negative affect (Anderson et al., 2019). Intolerance of uncertainty interferes with problem solving abilities (Patrick, 2016) and is a risk factor for deceased mental health, including issues such as pathological anxiety and depression (Shihata et al., 2016). This is particularly problematic because frequently a college students’ academic life is filled with uncertainty. For example, students may be uncertain about the content or difficulty of an exam, or uncertain about their instructors’ expectations for a term paper. The most recent meta-analysis examining rates of intolerance of uncertainty among college students suggests an increase in intolerance of uncertainty from 1994 to 2014 (Carleton et al., 2019).

When individuals experience discomfort associated with uncertainty, they are motivated to search for ways to reduce their discomfort, and one such strategy is procrastination. Students who report high intolerance of uncertainty experience more negative affect, including anxiety (Jensen et al., 2016). Students can attempt to reduce their uncertainty, and subsequently their discomfort, through a number of strategies, such as information seeking (Chowdhury et al., 2011). One harmful way that students cope with this uncertainty and stress is to procrastinate or attempt to avoid the task that is making them anxious, which can improve their mood temporarily (Sirois, 2023); this, at least partially, which explains why students high in intolerance of uncertainty are more likely to procrastinate (Fourtounas and Thomas, 2016).

Another method, or strategy, that individuals use to reduce the negative affect (i.e., anxiety and/or stress) associated with uncertainty is to seek social support from others. For example, intolerance of uncertainty in females is associated with more self-reported emotional support seeking and venting (Doruk et al., 2015). Moreover, previous literature finds that perceptions of social support moderate the association between amygdala reactivity to threatening stimuli and trait anxiety (Hyde et al., 2011), thus, highlighting the benefits of social support. However, the effectiveness of social support in reducing negative affect may differ based on the type of experience which is evoking uncertainty. For example, literature investigating uncertainty after women have been diagnosed with ovarian cancer suggests that social support does not buffer the association between intolerance of uncertainty and mental health symptoms. One plausible explanation is that some uncertain experiences are more distressing than others, and less benefited by social support (Hill and Hamm, 2019).

### Social support, academics, and procrastination

Previous literature suggests that perceptions of social support are critical to the educational experience, particularly among students and emerging adults. These emerging adults must take on more responsibility, such as financial responsibility (Marchant and Harrison, 2020) as they gain independence. Social support from various sources, including family, friends, and special others, can promote a sense of autonomy, motivation, positive self-evaluation, and mental health (Wang and Eccles, 2013; Feeney and Collins, 2015). The protective nature of social support is particularly important among college students. The undergraduate years tend to be a time of stressful transition for students as they grapple with social and academic integration (Lotkowski et al., 2004). Literature suggests that perceived social support can function as a protective mechanism by fostering positive emotional experiences, promoting self-regulation, and enhancing coping strategies (Feeney and Collins, 2015). Importantly, the psychological processes resulting from social support have been found to relate to procrastination, such that increased self-regulation, coping, and motivation have been linked to lower levels of procrastination (Pychyl and Flett, 2012; Feeney and Collins, 2015). Additionally, research suggests that working with others in groups is related to less procrastination and less negative affect (Koppenborg and Klingsieck, 2022). Engaging with social support as a mechanism to cope with negative affect can provide students with an alternative to maladaptive forms of coping, like procrastination.

Previous research has suggested a negative association between social support and academic procrastination (Ferrari et al., 1998). However, research examining the relationship between specific types of social support (e.g., friends and/or family) and academic procrastination among college students is limited. The most recent research suggests that family’s supportive attitudes towards academics is related to less procrastination in students (Borekci and Uyangor, 2018). Literature exploring the differences in academic procrastination between support from friends and family suggests that individuals who reported greater procrastination also reported greater dissatisfaction with their families and relied more on their peers for support. Additionally, peer support is negatively associated with procrastination (Handayani et al., 2021). Thus far, to our knowledge, no research has compared how different sources of social support (i.e., family, friends, special others) are linked to procrastination.

The problem of procrastination gets more complicated when considering gender and race. While intolerance of uncertainty in females is associated with more self-reported emotional support seeking and venting (Doruk et al., 2015), males and members of racially marginalized communities are more likely to report procrastination (Sabnis et al., 2022). Compounding this problem, these populations (i.e., males and members of racially marginalized communities) are also more likely to report experiencing less social support (Hefner and Eisenberg, 2009). For this reason, race and gender were used as controls in our analyses.

### The current study

The current study aims to explore sources of support (i.e., family, friends, and special others) and how they relate to college students’ procrastination. By investigating the different sources of support, this study investigates which sources of social support may be most effective in buffering the effects of intolerance of uncertainty and reducing procrastination among college populations. We hypothesized greater levels of family, friend, and special person support would each mitigate the association between intolerance of uncertainty and procrastination, such that higher levels of support, the impact of intolerance of uncertainty on procrastination would be reduced.

This study centered on procrastination of undergraduate students, who tend to report more procrastination than the general population (Steel, 2007). In this study, we included university students from a Hispanic Serving Institution, which is one of the populations that tend to be more at risk of procrastination (Sabnis et al., 2022) and also tend to be understudied.

## Methods

### Participants

This study consisted of 394 undergraduate participants, all taking an online Introduction to Psychology course in the year 2021 at a Southern California university. All participants taking the course were required to satisfy four units of research credit. Participants had the option of participating in a variety of ongoing studies at the university or writing short papers to satisfy the research requirement, and students who chose to participate in this study received two units of research credit. Participants self-identified as 58.5% female, 37.4% male, and 4.1% self-identified as other. The average age was 19.45, (*SD* = 1.94, Range = 18–35). Regarding race demographics, the participant sample was racially diverse and consisted of 39.1% Latinx, 36.7% Asian, 6.5% White, 6.5% self-identified other, 4.3% Middle Eastern, and 2.2% African American participants. The sample also consisted of 50.3% freshmen, 23% sophomores, 19.07% juniors, and 5.8% seniors. The sample size for this study was determined using G*Power, version 3.1 (Faul et al., 2009). No participant exclusion criteria exist for this study.

### Design

The data used in this study is cross sectional. When data collection was completed, survey items that needed to be reverse-coded were transformed, and then survey items were scored according to survey instructions. Skewness and kurtosis of variables were assessed and deemed acceptable. Data analysis, including regression modeling and the generation of the correlation table was conducted using R version 4.4.1 (R Core Team, 2024).

### Measures

#### Trait uncertainty

Trait uncertainty was measured using the 12-item Intolerance of Uncertainty Scale (IUS; Carleton et al., 2007). The IUS is measured on a Likert scale ranging from “1” (not at all characteristic of me) to “5” (entirely characteristic of me) and is computed by summing the item scores. Higher scores indicate greater trait uncertainty. An example item from the scale is “Unforeseen events upset me greatly.” In this study, a Cronbach alpha value for the IUS was assessed and demonstrated good reliability (*α* = 0.87).

#### Pure procrastination scale

Trait procrastination was measured using the Pure Procrastination Scale (PPS; Steel, 2010). The PPS consists of 12 items and is measured on a Likert scale ranging from “1” (Not at All) to “5” (Extremely) and is computed by averaging the scores. Higher scores indicate greater procrastination. The items measuring procrastination reflect a tendency to delay tasks. An example item from the PPS is “Even after I make a decision, I delay acting upon it.” In the current study. In this study, a Cronbach alpha value for the PPS was assessed and demonstrated excellent reliability (*α* = 0.91).

#### Perceived social support

Perceived social support was measured using the Multidimensional Scale of Perceived Social Support (MPSS; *α* = 0.91 Zimet et al., 1988). The MPSS is measured on a Likert scale ranging from “1” (Very Strongly Disagree) to “7” (Very Strongly Agree), and consists of 3 subscales, including family, friends, and special person perceived social support. Special person social support can be interpreted to mean a significant other or someone emotionally special to the individual. Scores are computed by averaging the items corresponding to subscale groups. Higher scores indicate more perceived social support. An example item from the MPSS is “My family really tries to help me.” In this study, a Cronbach alpha value for the MPSS was assessed and demonstrated excellent reliability (*α* = 0.91).

### Procedure

Participants were recruited from a participant pool at a Southern California university. Participants were administered the consent form and survey items via an emailed Qualtrics survey link consisting of personality measures and procrastination habits and were compensated with 2 units of class credit. Data collection took place during the Fall of 2021.

## Results

**Figure 1:**
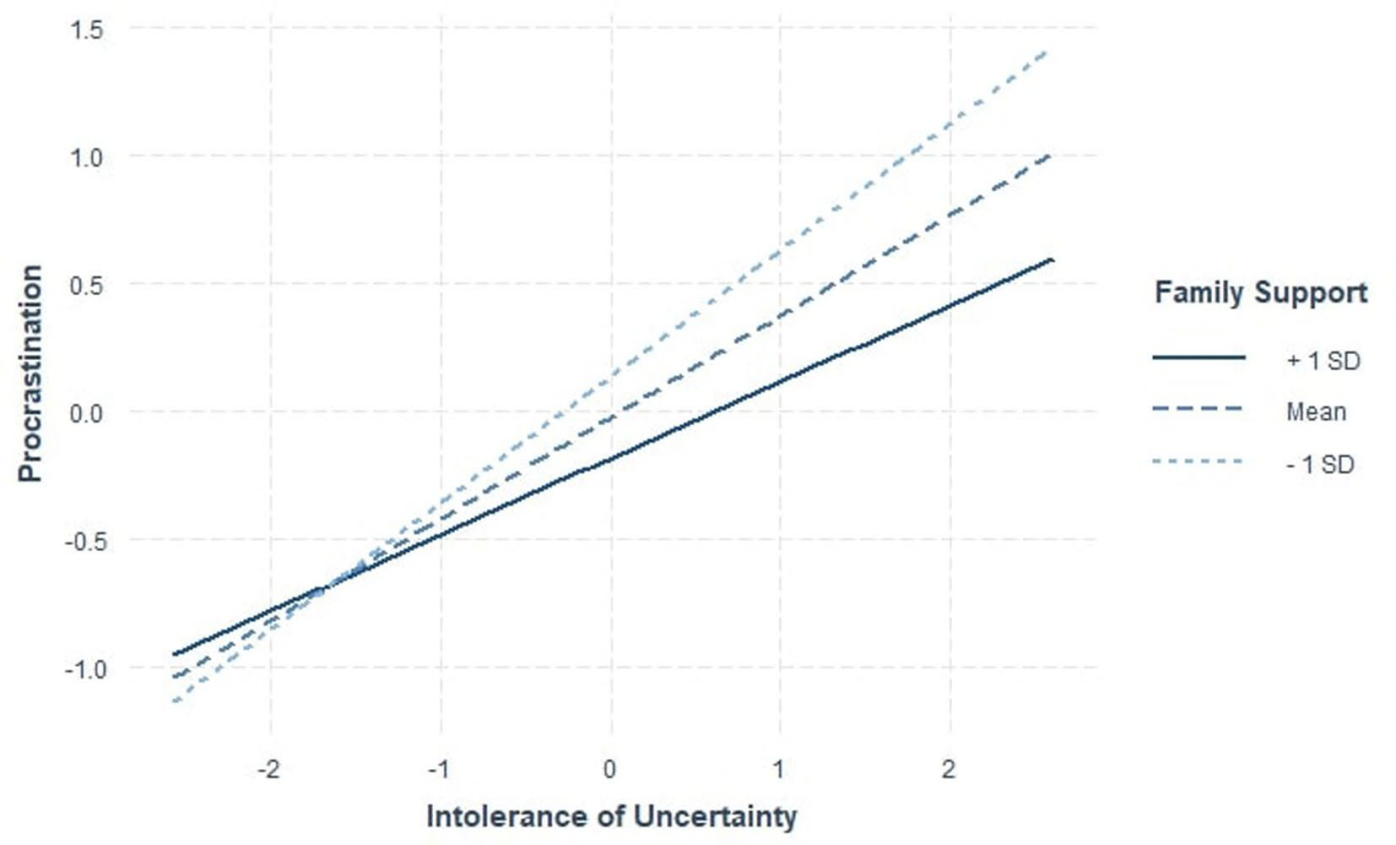
Intolerance of uncertainty and family social support interaction. The figure depicts the significant interaction between family social support and intolerance of uncertainty predicting procrastination. *N* = 389.

Descriptives are presented in Table 1. Correlation analyses revealed a significant negative relationship between procrastination and family and friend support. Special person social support was not significantly related to procrastination (see Table 1). OLS regression analyses were conducted to test the stated hypotheses. Five responses were excluded from data analysis due to missing participant data. All analyses controlled for race (i.e., Asian and Latinx) and gender (i.e., male and female). In the first model, intolerance of uncertainty, family social support, friend social support, and special person social support were tested as predictors of procrastination. In the second model, the moderating effects of social support on intolerance of uncertainty and procrastination were tested. Consistent with the study hypotheses, high levels of family support and friend support significantly predicted lower levels of procrastination. Additionally, a significant interaction emerged between intolerance of uncertainty and family social support in predicting procrastination, suggesting that at high levels of intolerance of uncertainty, high levels of family support were linked with lower levels of procrastination (see Figure 1). Inconsistent with our hypotheses, special person social support did not significantly predict levels of procrastination (see Model 1). Finally, no significant interactions were observed between procrastination and either friend social support or support from a special person support (see Model 2) (Table 2).

**Table 1:** Correlation table among key study variables.

Variable	*M*	*SD*	1	2	3	4	5	6	7	8
1. Procrastination	2.96	0.77	1							
2. IOU	2.94	0.72	0.42**	1						
3. Family support	4.60	1.65	−0.21**	−0.14**	1					
4. Friend support	5.37	1.4	−0.17**	−0.10*	0.35**	1				
5. Special Person support	5.14	1.7	−0.06	−0.07	0.40**	0.53**	1			
6. Latinx	0.39	0.49	0.04	0.01	0.06	0.02	0.06	1		
7. Asian	0.37	0.48	0.03	0.07	−0.12*	−0.05	−0.13**	−0.61*	1	
8. Gender	0.39	0.49	0.05	0.02	0.02	0.01	0.08	0.18**	−0.16**	1

* indicates *p* < 0.05, ** indicates *p* < 0.01. M and SD represent mean and standard deviation, respectfully. IOU, intolerance of uncertainty. Latinx is dummy coded 1 (Latinx) and 0 (not Latinx), Asian is dummy coded 1 (Asian) and 0 (not Asian), and Gender is dummy coded 0 (Male) and 1 (Female). *N* = 389.

**Table 2:** Regression with predictors and controls.

Model	Predictor	Beta	*SE*	*p*	95% CI		df	*R* ^2^
					LL	UL		
1	IOU	0.39	0.05	0.001	0.30	0.48		
	Family support	−0.15	0.05	0.002	−0.25	−0.05		
	Friend support	−0.11	0.05	0.04	−0.21	−0.005		
	Special person support	0.08	0.05	0.15	−0.03	0.19		
	Latinx	0.14	0.12	0.24	−0.10	0.38		
	Asian	0.07	0.12	0.59	−0.18	0.31		
	Gender	0.08	0.09	0.41	−0.11	0.26		
							389	0.23
2	IOU	0.39	0.04	0.001	0.30	0.49		
	Family support	−0.16	0.05	0.002	−0.26	−0.06		
	Friend support	−0.12	0.05	0.03	−0.22	−0.009		
	Special person support	0.07	0.05	0.18	−0.04	0.18		
	Latinx	0.15	0.12	0.21	−0.09	0.39		
	Asian	0.08	0.12	0.50	−0.16	0.33		
	Gender	0.07	0.09	0.47	−0.11	0.25		
	IOU*Family support	−0.01	0.05	0.04	−0.19	−0.006		
	IOU*Friend support	−0.07	0.06	0.19	−0.18	0.04		
	IOU*Special person support	0.03	0.06	0.60	−0.08	0.14	389	0.23

IOU, intolerance of uncertainty, Latinx is dummy coded 1 (Latinx) and 0 (not Latinx), Asian is dummy coded 1 (Asian) and 0 (not Asian), and Gender is dummy coded 0 (Male) and 1 (Female). *N* = 389.

## Discussion

In the current study, family support, but not friend support or special other support, moderated the relationship between intolerance of uncertainty and procrastination among college students. This suggests that family support could be helpful for mitigating procrastination, particularly for those at risk, namely students high in intolerance of uncertainty. Study findings suggest that family support may operate as a protective factor, reducing the risk of intolerance of uncertainty on procrastination among college students. These findings are consistent with prior literature (Borekci and Uyangor, 2018), suggesting that social support from families is protective in academic environments. Importantly, study findings suggest that support from families may provide college students with valuable resources for coping with their emotional challenges and avoiding maladaptive forms of coping like procrastination.

Regarding insignificant study findings, friend support did not moderate the relationship between intolerance of uncertainty and procrastination. This was unexpected, given that previous research has suggested peer support is related to less procrastination (Handayani et al., 2021). Given that data collection for this study took place during remote instruction, it is possible students may not have had access to friend and peer social networks. Additionally, it is possible that support from special others did not moderate the relationship between intolerance of uncertainty and procrastination, likely because the quality of support from significant others may be different for emerging adults than in older adult populations.

Previous work has suggested that college is a difficult transition time for young adults, who must grapple with increased responsibility and autonomy (Marchant and Harrison, 2020), and leads to an increase in procrastination risk factors, including intolerance of uncertainty (Carleton et al., 2019). These challenges make it more likely for students to rely on maladaptive coping strategies like procrastination, which may explain why self-ratings of procrastination are higher in college students than the general population (Steel, 2007). The results of this study suggest that perceived family support can be helpful for mitigating procrastination. These findings have direct application to university staff, academic counselors, and psychological counselors, who should recognize that family support is critical to students; therefore, they should encourage students to leverage family support as a resource. For example, counselors could encourage students to communicate with their parents when they struggle with academic challenges, when they experience difficulty tolerating uncertainty, and/or when they suffer negative emotional affect such as anxiety.

### Strengths and limitations

As with any study, there are strengths and limitations. In this study, one of the strengths is that it was among the first of its kind to investigate family, friends, and special persons as sources of social support in procrastination. Additionally, this study sample was racially diverse, consisting heavily of Latinx and Asian students, which are both growing populations in higher education and understudied populations. For our limitations, this sample consisted primarily of freshmen and sophomores, and it is possible these underclassmen had yet to develop friend groups and friend support networks so early in their college experience. It is also probable that students simply had greater and easier access to family support, given that this data was collected during the COVID-19 pandemic when students were still participating in remote learning.

Finally, it is not clear if underclassmen benefit from family support more than their upperclassmen peers. The early years of college involve challenges such as transitioning from high school and learning to navigate college, and this can be a highly stressful time for students (Lotkowski et al., 2004). For example, new college students must learn to independently manage their studying, especially in terms of balancing their course load and working schedule (Cleary et al., 2011). Consequently, underclassmen may benefit more from social support than their upperclassmen peers who have already successfully integrated into the college experience.

### Future directions

Future studies should probe the relative benefits of specific family member support and its quality in relation to procrastination. Additionally, links between procrastination and support given from others that fall outside of the categories of family, friends, and significant partners, such as mentors and advisors, warrant investigation. These findings have important implications for college programs seeking to reduce students’ procrastination by advising students to leverage familial support as a protective factor when students struggle with uncertainty, anxiety, or stress.

## Data Availability

The raw de-identified data supporting the conclusions of this article will be made available by the authors upon request, without undue reservation.
